# Acute Pericarditis After Percutaneous Coronary Intervention

**DOI:** 10.7759/cureus.95244

**Published:** 2025-10-23

**Authors:** Ahmed Smman, Infanta Raffael

**Affiliations:** 1 Cardiology, Barnet Hospital, London , GBR; 2 Medicine, Royal Free Hospital, London, GBR

**Keywords:** acute pericardial effusion, acute pericarditis, cardiac chest pain, colchicine therapy, electrocardiography (ecg), non-steroidal anti-inflammatory drug (nsaid), pericardial friction rub, primary pci, st-elevation myocardial infarction (stemi), transthoracic echocardiogram

## Abstract

Pericarditis may be a complication of percutaneous coronary intervention (PCI), and should be considered as a differential for persistent chest pain following a PCI.

In this report, we present a male in his 60s, who developed pericarditis after a primary PCI for an anterior ST elevation myocardial infarction (STEMI). He had an uncomplicated PCI to left anterior descending artery, and was discharged as per standard procedure 48 hours post-procedure. On that same evening, the patient re-presented to hospital with different chest pain and fever. Electrocardiogram (ECG) revealed anterior ST elevation.

A provisional diagnosis of pericarditis was made in view of elevated inflammatory markers, a recent Mycoplasma infection with fevers, positron emission tomography-computed tomography (PET-CT) findings, as well as pericardial chest pain. A three-month course of colchicine was initiated. Two weeks after discharge, he was readmitted with non-resolving fevers. Echocardiogram (ECHO) and CT thorax revealed a 26mm pericardial effusion. This was not drained, as he had no signs of tamponade. He was treated as possible infective pericarditis with intravenous (IV) teicoplanin and piperacillin-tazobactam, which was stepped down to oral co-trimoxazole on discharge. Three weeks after completing the oral antibiotics, he was re-admitted again with fevers and chest pain. He was restarted on antibiotics and heart failure medications were up-titrated. He was continued on colchicine and started on ibuprofen. Due to the recurrent episodes of pericarditis, he was also commenced on a weaning course of prednisolone. The patient's symptoms subsequently improved after treatment, and he is awaiting follow-up with his Cardiologist, alongside a repeat ECHO prior to the appointment.

## Introduction

Pericarditis is inflammation of the membrane surrounding the heart, and usually manifests as sharp, stabbing central chest pain; other symptoms may include shortness of breath, fever, or fatigue [1]. Causes include viral infections (in developed countries), tuberculosis (in developing countries), autoimmune diseases, myocardial infarction, cardiac surgery, or idiopathic [2]. Diagnosis is based on two of the following: chest pain, new widespread ST-elevation or PR depression on electrocardiogram (ECG), pericardial friction rub and pericardial effusion [3]. 

Pericarditis may occur after percutaneous coronary intervention (PCI). Proposed mechanisms of post-PCI pericarditis include damage to the pericardial cells, which may lead to activation of immune responses and inflammation [1]. 

Mainstays of treatment include non-steroidal anti-inflammatory drugs (NSAIDs) or colchicine [3]. In the United Kingdom, there is an annual incidence of approximately 27.7 individuals per 100,000, and recurrence can occur in up to 30% of cases after the first episode [4].

## Case presentation

A male in his 60s, otherwise well, presented to hospital with central, non-radiating chest pain and nausea. Examination was unremarkable. ECG showed ST-elevation in V1-V4 and troponin measured at 71. He underwent urgent coronary angiography demonstrating thrombotic left anterior descending (LAD) occlusion with no by-stander disease and received a drug-eluting stent. Transthoracic echocardiography (TTE) showed left ventricular ejection fraction (LVEF) ~45% with apical regional wall-motion abnormality. He was discharged on aspirin, prasugrel, atorvastatin, bisoprolol, ramipril and lansoprazole with standard post-PCI advice. The evening of discharge, he re-presented with pleuritic chest and shoulder pain and low-grade fever, qualitatively different from his initial ischaemic pain. ECG changes were consistent with resolving anterior ST elevation myocardial infarction (STEMI) without new ischaemia and auscultation revealed a pericardial rub. Computed tomography pulmonary angiography (CTPA) excluded pulmonary embolism. TTE and computed tomography (CT) thorax showed a ~20 mm pericardial effusion without tamponade. Given a recent Mycoplasma infection and pericarditic pain, Dressler's syndrome was suspected; colchicine for three months was commenced alongside a 19-day doxycycline course. Twelve days later he re-attended with bilateral shoulder pain and fever. Inflammatory markers were significantly elevated; repeat focused TTE again showed a small-moderate effusion and LVEF 40-45% with apical hypokinesis. Broad septic screening was initiated and empiric intravenous (IV) piperacillin-tazobactam and teicoplanin were started after microbiology discussion in order to empirically manage possible infective pericarditis. CT and positron emission tomography (PET) revealed hypermetabolic pericardial effusion with surrounding fat stranding, supporting active pericarditis. Given absence of tamponade and an unfavourable subxiphoid window, pericardiocentesis was deferred. Cultures remained negative and inflammatory markers down-trended. Three weeks after completing oral co-trimoxazole, he re-presented with recurrent fevers and pericarditic chest pain. IV antibiotics were re-instituted while repeating cultures; anti-inflammatory therapy was optimised. Given recurrent symptoms despite NSAID/colchicine and repeatedly negative cultures, a cautious prednisolone wean was initiated with subsequent improvement. 

A six-week follow-up appointment was arranged with the Cardiologist, along with a TTE prior to his appointment to visualise the progress of the effusion. He was advised not to partake in strenuous activities for two weeks, such as lifting, and given advice to return to hospital should he have further episodes of chest pain or shortness of breath.

**Figure 1:**
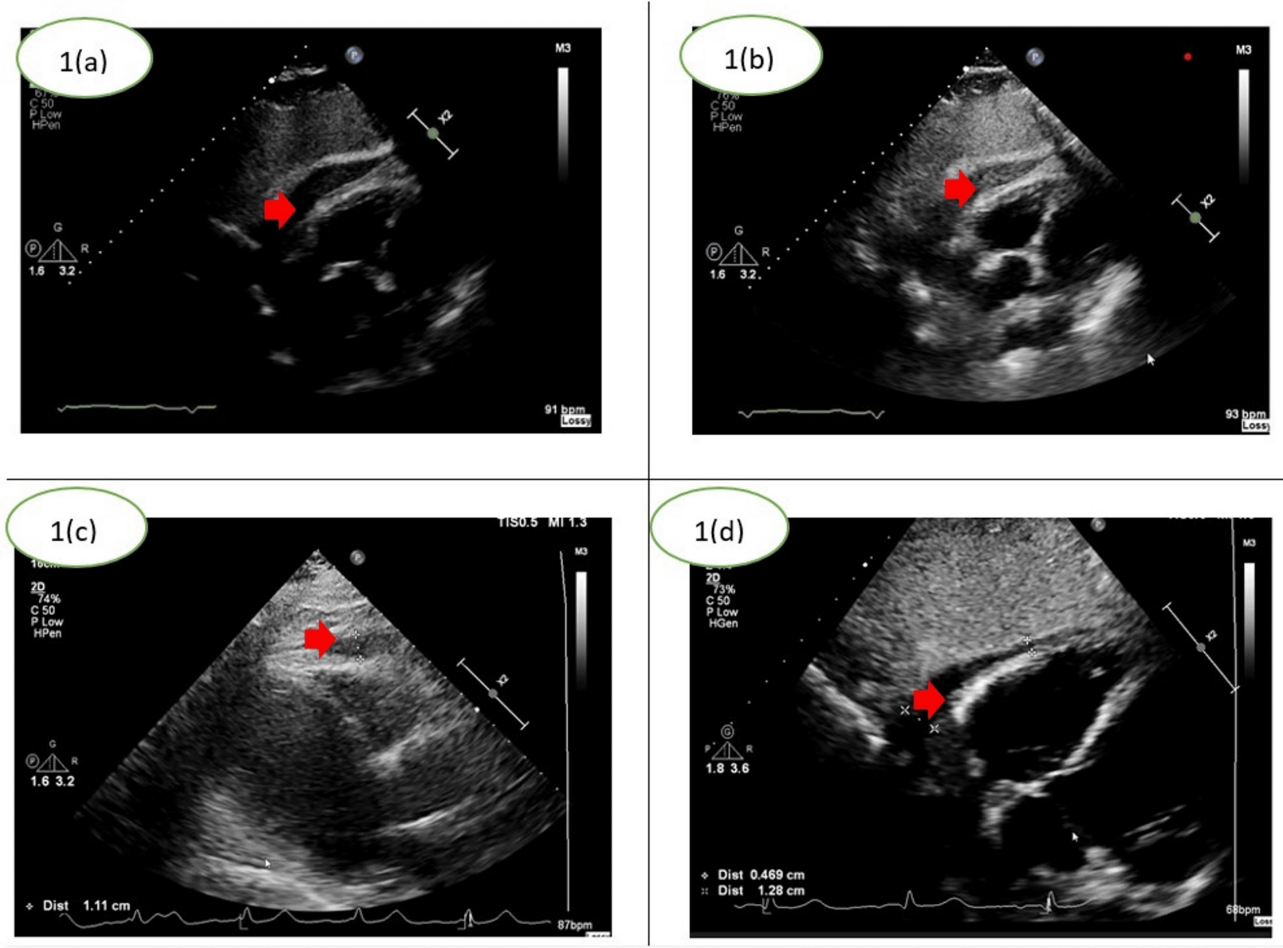
Subcostal transthoracic echocardiography (TTE) views of the pericardial effusion. 1(a)- Initial TTE showing small pericardial effusion after percutaneous coronary intervention. 1(b)- Increase in effusion size on day 12 with associated pericarditic pain. 1(c) and (d)- Reduction in effusion after anti-inflammatory and corticosteroid therapy. Red arrows demonstrate the location of the pericardial effusion. TTE images above were obtained from patient records.

The following TTE images demonstrate the progression of the pericardial effusion during his hospital stay (Figure 1A-1D). Laboratory results including troponin, C-reactive protein, and white cell count are summarised in Table 1. It was noted that the patient's symptoms improved as the effusion size reduced as shown in Figure 1C, 1D.

**Table 1:** Blood test results during admission with normal reference ranges. Laboratory results showing admission, peak, and discharge values with normal reference ranges obtained from patient records.

Markers	On admission	Peak	On discharge	Normal ranges
Troponin T	71	71	28	<14 ng/L
C-reactive protein	90.5	257.5	12.5	<3 mg/L
White cell count	7.42	15.88	8.7	4.5-11 x 10^9/L
Blood cultures	No growth after 5 days			

The first differential diagnosis considered was another myocardial infarction. However, the declining troponin levels, absent dynamic changes in ECGs, alongside the different character of chest pain ruled this out. CTPA was negative for pulmonary embolism, and TTE ruled out cardiac tamponade. There were no clinical signs of any musculoskeletal injuries.

## Discussion

Post-cardiac injury syndrome (PCIS) is a major cause of pericarditis. Damage to the pericardial cells can be caused by surgery, blunt trauma, myocardial necrosis, or iatrogenic [1]. Although the damage caused by PCI may be regarded as minimal, this can lead to activation of immune responses and inflammation. Early infarct-associated pericarditis occurs after five days post-myocardial infarction, whereas late post-myocardial infarction pericarditis (Dressler’s syndrome) occurs two to eight weeks after infarction [1]. In developed countries, the incidence of PCIS is less than 5%, deeming it to be rare [5].

A similarly published case in 2021 involved a gentleman in his 50s, who was admitted for an elective PCI of a chronic total occlusion to the LAD artery. Six hours post-procedure, the patient developed sub-sternal chest pain, worsened by lying down. Repeat angiogram did not show any new findings, and ECG revealed ST-elevation in the anterior and lateral leads. TTE revealed a small pericardial effusion, and a 15-day course of ibuprofen was commenced. A three-fold reduction in C-reactive protein (CRP) was observed after three days of treatment [6].

Another interesting case in 2023 demonstrates a gentleman in his late 70s, who developed constrictive pericarditis after coronary artery perforation during PCI. He was initially admitted due to chest pain and underwent coronary angiogram, which revealed diffuse triple-vessel disease, with right coronary artery (RCA) chronic total occlusion (CTO). PCI at the RCA CTO lesion was complicated by perforation during right ventricular (RV) branch wiring proximal to the lesion. After prolonged balloon inflation, it seemed that the perforation had resolved. A follow-up TTE at one week showed no visible pericardial effusion, and the patient was discharged. Seven months later, the patient re-presented to hospital with dyspnoea and abdominal distention. Coronary angiogram revealed no new lesions, and TTE revealed a small pericardial effusion. He was therefore discharged on prednisolone and colchicine. Two weeks later, the patient went back to hospital with worsening abdominal distention and dyspnoea. TTE showed worsening pericardial effusion and constrictive physiology. The patient became haemodynamically unstable with increasing oxygen requirement, and had to undergo radical pericardiectomy. A thickened pericardium (5mm) was found intraoperatively, and a dark bloody pericardial effusion was drained. He had no complications post-operatively, and was discharged on diuretics with symptoms much improved [7]. 

Prompt recognition alongside appropriate imaging enables patients to receive treatment in a timely manner. This can prevent complications, such as cardiac tamponade and constrictive pericarditis, which in turn improves patient outcomes. 

## Conclusions

Mycoplasma infection, although rare, can be a cause of pericarditis. It was difficult to differentiate whether the pericarditis was caused by the Mycoplasma infection or post PCI in this case study, as both factors were involved. It may be worth considering delaying discharge after a PCI if the patient is still having signs of infection to rule out pericarditis. However, this decision should be individualised, based on infection severity, comorbidities, and risk of complications. It is vital to be vigilant and consider PCI complications for patients who present with atypical chest pain.
